# Adolescents’ perceived barriers to accessing sexual and reproductive health services in California: a cross-sectional survey

**DOI:** 10.1186/s12913-021-07278-3

**Published:** 2021-11-22

**Authors:** Martha J. Decker, Tara V. Atyam, Catherine Gilmore Zárate, Angela M. Bayer, Consuelo Bautista, Melissa Saphir

**Affiliations:** 1 Department of Epidemiology and Biostatistics, University of California, San Francisco, San Francisco, CA USA; 2 Philip R. Lee Institute for Health Policy Studies, University of California, San Francisco, 490 Illinois St, San Francisco, CA 94107 USA; 3 Bixby Center for Global Reproductive Health, University of California, San Francisco, San Francisco, CA USA; 4 California Department of Public Health, Maternal, Child and Adolescent Health Division, 1615 Capitol Ave, MS 8300, P.O. Box 997420, Sacramento, CA 95899-7420 USA

**Keywords:** Adolescents, Sexual health, Sex education, Reproductive health services, California, Barriers to care, Burden of barriers, Confidentiality

## Abstract

**Background:**

Adolescents may forego needed sexual and reproductive health (SRH) services due to a variety of concerns and barriers. The purpose of this study is to compare adolescents’ perceptions of these barriers by participant characteristics including race/ethnicity, gender, sexual orientation, housing situation, and sexual experience.

**Methods:**

Adolescents in a California-wide sexual health education program completed an anonymous survey at baseline (*N* = 10,015) about perceived barriers to using SRH services. Logistic regression analyses that accounted for the clustered data structure assessed differences by gender, age, sexual orientation, race/ethnicity, living situation, and sexual experience.

**Results:**

The majority of participants were Hispanic/Latino (76.4%) with an average age of 14.9 years, and 28.8% had sexual experience. Half of the youth reported concerns about test results (52.7%), cost of services (52.0%), and confidentiality of services (49.8%). When controlling for other characteristics, youth identifying as transgender/non-binary/multiple genders had the highest odds of perceiving cost (odds ratio (OR) 1.89) and confidentiality (OR 1.51) as barriers. Increasing age was associated with decreasing odds of all barriers. Sexual orientation was a consistent predictor, with LGBQ+ youth having higher odds of perceiving test results (OR 1.21), cost (OR 1.36), and confidentiality (OR 1.24) as barriers. Asian or Pacific Islander/Native Hawaiian youth had higher odds of perceiving test results (OR 1.68) and cost (OR 1.37) as barriers. In contrast, Black youth had lower odds of reporting cost (OR 0.65) and confidentiality (OR 0.77) as barriers. Younger respondents and youth who identified as female, transgender/non-binary/multiple genders, LGBQ+, and Asian or Pacific Islander/Native Hawaiian had higher odds of reporting five or more barriers compared to reference groups.

**Conclusions:**

The majority of adolescents face barriers to accessing appropriate SRH services, with females, gender-minority youth, younger adolescents, LGBQ+ youth, and Asian and Pacific Islander/Native Hawaiian youth more likely than others to report barriers. Access to SRH services can be improved through strengthening linkages between clinics and SRH education programs, providing youth-friendly clinical services, and ensuring youth have sufficient information, skills, and support to access care.

**Trial registration:**

Approved by California Health and Human Services Agency’s Committee for the Protection of Human Subjects [12-08-0658, 11/30/2017].

**Supplementary Information:**

The online version contains supplementary material available at 10.1186/s12913-021-07278-3.

## Background

Adolescents may forego needed sexual and reproductive health (SRH) services due to a variety of concerns and barriers. Previous research has identified common barriers to SRH services among adolescents that include confidentiality, stigma, embarrassment, and fear [1, 2]. Structural challenges such as cost, location, transportation, and limited scheduling also may reduce adolescents’ access to services [3]. Additionally, adolescents often cite unfriendly or judgmental interactions or distrust of providers as a reason for not seeking sexual health services [4, 5].

Certain groups of adolescents face greater barriers to accessing services than others, with disparities in access based on gender, race and ethnicity, and sexual orientation, among other characteristics. While concerns about confidentiality and embarrassment are common across youth of all genders and backgrounds, one study found that female youth who were concerned about confidentiality, particularly related to their parents, were less likely to receive contraceptive services [6]. A qualitative study of young men identified a range of challenges, including the perception that SRH services were not designed for them [7]. Studies of lesbian, gay, bisexual, transgender, and queer (LGBTQ) youth have found respectful providers to be a priority and that transgender youth were more likely to postpone or not get care due to provider discrimination [8, 9]. One large survey of female youth found that sexual minority females were less likely to access routine screenings such as sexually transmitted infection (STI) testing and Pap smears, although bisexual females were significantly more likely to have had a positive STI test compared to heterosexuals or lesbians [10]. Similarly, sexual minority females are more likely to become pregnant during adolescence than heterosexual females [11].

Youth of certain racial and ethnic backgrounds also experience disparities in access as well as concerns about stigma and discrimination [1, 12]. Prior research of gendered racism, discrimination at the intersection of race/ethnicity and gender, has recognized the impact of contraceptive mistrust and stereotypes regarding sexual and reproductive behaviors among Black and Latina women [13]. One qualitative study of Latina youth identified barriers at the sociocultural, relational, and individual levels, with the most common being parental attitudes toward SRH access [14]. Little research has focused on other racial and ethnic groups, including Asian Americans and Native Americans. A quantitative analysis found that Native Americans at every age, including adolescence, were less likely to use contraceptive services than White women, but more likely to use services for HIV and STIs [15]. One qualitative study of Asian American young women recognized concerns about confidentiality and cultural stigma as barriers to using SRH services [16]. The intersection of these different demographic characteristics may also amplify concerns among certain groups. For example, Latino youth living in rural areas often worry about privacy due to small and tight-knit communities and have limited services available [17].

Youth in vulnerable or unstable living situations may face additional barriers to accessing SRH services including a lack of a regular healthcare provider and unfamiliarity with local services. One survey of homeless and runaway youth found that transportation, cost, and lack of insurance were common barriers to seeking healthcare services [18]. One small qualitative study of youth leaving foster care found that confidentiality concerns related to their foster parents limited their interaction and communication regarding SRH needs with their healthcare providers [19].

These barriers to SRH care perceived by adolescents fall into five key domains that Penchansky and Thomas identified as affecting access to health care: affordability, availability, accessibility, accommodation, and acceptability [20]. While these domains represent essential structural and individual considerations, it is also important to examine how experiencing multiple barriers affects youth. To date, minimal research has evaluated the impact of multiple barriers on youth when accessing SRH services. One study of women accessing abortion care found that experience of multiple barriers can have a compounding effect, resulting in negative consequences for the patient [21]. The burden of these barriers may be interconnected and cyclical. A secondary analysis of a survey of adults conducted in 11 countries including the United States found that vulnerable groups are more likely to experience multiple barriers in accessing care [22]. The cumulative result of multiple barriers and the related disparities in access has not been assessed among adolescents.

While there have been studies of barriers to adolescent access to SRH services in other countries, the majority of studies in the United States have been qualitative, with small samples drawn from a specific population of interest [23–25]. Few have compared these barriers across different populations or assessed which populations have the highest burden of barriers. This study builds on Penchansky and Tomas’s theory of access by specifically incorporating a health disparities and equity lens [26]. The purpose of this study is to quantitatively assess adolescents’ perceptions of barriers to accessing SRH services by demographic and behavioral characteristics prior to receiving sexual health education.

## Methods

### Program setting

All participants in California’s Personal Responsibility Education Program (CA PREP) were asked to complete a baseline survey at program entry. This program is a federally funded initiative administered through the California Department of Public Health’s Maternal, Child and Adolescent Health Division. CA PREP aims to reduce unintended pregnancy and STIs among California’s adolescents by providing them with comprehensive, medically accurate, and unbiased SRH education, in compliance with the 2016 California Healthy Youth Act [27]. Twenty-two agencies serving 20 high-need counties encompassing urban, suburban, and rural areas across California implemented the program in 2018-2019 using one of four evidence-based curricula [28]. Program eligibility is based on several county-level indicators including, but not limited to, adolescent birth rate, percentage of adolescent repeat births, gonorrhea index rate, and percentage of youth living in concentrated poverty and/or racially isolated areas [29]. Program delivery is prioritized for vulnerable youth, such as those in foster care and juvenile justice systems, migrant youth, homeless and runaway youth, youth with special needs, and LGBTQ youth. A majority of program recipients are Latino, reflecting the fact that the majority of school-aged children (55%) in California are Latino, with higher percentages in the southern part of the state [30]. PREP serves youth in more than 300 unique sites, including traditional high schools, alternative or continuation schools, shelters and transitional housing, middle schools, juvenile justice facilities, community-based organizations, foster care and clinics.

### Participants and procedures

Confidential paper surveys, in English and Spanish, were administered by trained facilitators from implementing agencies to youth at the beginning of the program. Prior to the survey, all participants completed an assent/consent form. In addition, passive parental or guardian consent, meaning parents or guardians needed to sign and return the consent form for refusal, was used per state guidelines for sexual health education [27]. Between July 1, 2018 and June 30, 2019, 13,564 youth participated in the program and 10,558 completed the voluntary, anonymous survey (77.8%). Youth ages 20 and 21 or older (*n* = 93) and those who skipped the barriers questions (*n* = 450) were dropped from the analysis for a final sample size of 10,015 adolescents. The 22-question survey was approved by the Committee for the Protection of Human Subjects [12-08-0658] of the California Health and Human Services Agency.

### Dependent variables: perceived barriers

Measures were based on Penchansky and Tomas’s dimensions of access to health care [20, 31] and refined to reflect prior research on adolescent barriers to SRH services (Table 1). Although the original theory did not include awareness as a dimension, subsequent research has recommended this addition [32]. Similarly, concern about test results was added as a barrier in the accommodation dimension based on previous research [11, 33]. Prior to implementation, the survey was pilot tested with youth and health educators, and wording was revised as needed.

**Table 1:** Dimensions of access to care

Penchanksy and Thomas’s dimensions of access	Survey item
**Availability**: volume and type of services to meet the clients’ needs	**Lack of access**: It would be easy for me to go to a clinic^1^
**Accessibility**: location of the services and how easily the clients can reach the location, including cost and travel time	
**Accommodation**: how services are organized (including appointment systems, telephone services) and the clients’ perception of their appropriateness	**Confidentiality of services**: I would be worried about getting sexual health services because my parents/guardians, family, sexual partner, or friends may find out **Test results**: I would be worried about test results
**Affordability**: the prices of services and insurance requirements compared to the clients’ ability to pay	**Cost of service**: I would be worried about cost
**Acceptability**: clients’ preferences for the personal characteristics and attitudes of providers	**Judgment by staff**: I would be worried that clinic staff may judge or disrespect me **Discomfort talking with staff**: I would feel comfortable talking with clinic staff about my sexual health and questions
**Awareness:** client knowledge and awareness of existing services through effective communication and information strategies	**Lack of knowledge about visit**: I know what to expect if I go to a clinic

^1^A single question operationalizes the dimensions of availability and accessibility, because they overlap conceptually

A previous question in the survey defined sexual health services as “a clinic or doctor in your community where teens can get sexual health information and services (such as condoms, birth control, pregnancy tests, and STI and HIV tests)”. Responses to four questions (about test results, cost, confidentiality, and judgment) were coded as follows: “agree” and “strongly agree” were coded 1, and “disagree” and “strongly disagree” were coded 0. Responses to three questions (about comfort talking to clinic staff, knowledge, and access) were reverse-coded, so that all questions represent a barrier to SRH clinic services: “disagree” and “strongly disagree” were coded 1 and “agree” and “strongly agree” were coded 0.

To operationalize burden of barriers, we first summed the seven dichotomous variables described above and examined the distribution of the resulting sum. A sharp drop-off in the distribution was observed at five perceived barriers, with only 10.0% perceiving five barriers and only 7.0% perceiving six or seven barriers. Because we were interested in severe burden of barriers, we transformed the sum into a dichotomous variable with five as the cutpoint. Participants who perceived five, six, or seven barriers were coded 1 (high burden of barriers,) and all others were coded 0 (low burden of barriers).

### Independent variables: participant characteristics

#### Gender

Respondents were asked to mark all gender responses that apply. Responses were coded as male, female, and transgender/non-binary/multiple genders.

#### Age

Respondents were asked to mark their ages from 10 to 21 years or older. Youth ages 20 and older were removed from the analysis. Age was entered as a continuous variable in the regression analyses.

#### Sexual orientation

Respondents were asked to mark all sexual orientation categories that apply. Responses were coded as straight/heterosexual or LGBQ+. The LGBQ+ category included “Gay,” “Lesbian,” “Bisexual,” “Queer,” “Questioning,” “Other,” and multiple responses.

#### Ethnicity and race

For ethnicity, respondents were asked “Are you Hispanic or Latino?” For race, respondents were asked to mark all race categories that apply. Response options were “White,” “Black or African American,” “Asian,” “Native Hawaiian or Pacific Islander,” and “American Indian or Alaska Native.” Due to small numbers, “Asian” and “Native Hawaiian or Pacific Islander” responses were combined into one category, Asian or Pacific Islander/Native Hawaiian. Almost half (46%) of the participants declined to provide a race category. Of these, 96% identified as Hispanic; therefore, ethnicity and race responses were combined for the multivariate analysis. Those who said they were “Hispanic or Latino” were coded as Hispanic, regardless of any race they marked. Among those who were not Hispanic or Latino, those who marked a single race were coded as that race, while those who marked multiple races were coded as multiple.

#### Living situation

From a list of seven living situations, respondents were asked to select all that describe where they currently live*.* “In foster care, living with a family” and “In foster care, living in a group home” were coded as being in foster care. “In juvenile detention, jail, prison, or another correction facility, or under the supervision of a probation officer” was coded as juvenile justice facility. “Couch surfing or moving from house to house”, “Living in a place not meant to be a residence”, and “Staying in an emergency shelter, transitional living program, or motel” were coded as unstable housing. Respondents who did not mark any of the options provided were coded as having stable housing.

#### Sexual experience

Respondents were asked two questions about their sexual experience: “Have you ever had vaginal sex (penis in vagina)?” and “Have you ever had anal sex (penis in butt)?” Those who responded “Yes” to either or both of these questions were coded as having ever had vaginal and/or anal sex. All others were coded no.

### Analysis

To understand how youth differed in perception of barriers to SRH services, we first examined the dependent variables within each participant characteristic at baseline using one-way ANOVA with Bonferroni adjustment for multiple comparisons. Next, we conducted logistic regressions to calculate odds ratios for each participant subgroup while controlling for other demographic variables and sexual experience. We used the *melogit* command in Stata version 16 [31] to account for the non-independence of data collected from students clustered within cohorts and cohorts clustered within agencies [34]. In all analyses, *p* values of 0.05 or less were considered statistically significant.

## Results

### Participant characteristics

There were slightly more male (51.9%) than female respondents (46.8%), with 1.3% of youth identifying as transgender or non-binary or selecting multiple responses (Table 2). The sample was fairly evenly split between 10- to 14-year-olds (46.0%) and 15- to 19-year-olds (54.0%). The majority of participants identified as straight or heterosexual (84.4%), and 15.6% identified with LGBQ+ sexual orientations. The majority of participants identified as Hispanic or Latino ethnicity (76.4%), and the majority of these youth did not report their race. Among all participants, 46.1% did not identify a race, 25.9% identified as white, 8.2% as American Indian/Alaska Native, 7.7% as Black, 6.0% as Asian or Pacific Islander/Native Hawaiian, and 6.2% as multiple races. The majority (88.2%) reported living in stable housing. Much smaller percentages reported living in foster care (6.1%), juvenile justice facilities (3.7%), or an unstable housing situation (2.1%). Most participants (71.2%) had never had vaginal and/or anal sex. Youth with sexual experience tended to be older (43.7% of 15- to 19-year-olds were sexually active, compared to 11.4% of 10- to 14-year-olds) and male (34.9% of males were sexually active, compared to 22.2% of females and 28.8% of youth identifying as transgender, non-binary or multiple genders). Over half of youth (56.3%) had heard of a clinic where SRH services are provided.

**Table 2:** Participant characteristics

	Total (***N*** = 10,015)	Percent
**Gender**		
Male	5,166	51.9
Female	4,664	46.8
Transgender/non-binary/multiple genders	129	1.3
Age		
10-14 years	4,579	46.0
15-19 years	5,369	54.0
**Sexual orientation**		
Straight/heterosexual	8,140	84.4
Bisexual	666	6.9
Questioning	200	2.1
Gay/lesbian	179	1.9
Multiple/queer/other	458	4.8
**Ethnicity**		
Hispanic	7,546	76.4
Non-Hispanic	2,328	23.6
Race		
White	2,590	25.9
American Indian/Alaska Native	822	8.2
Black	766	7.7
Asian or Pacific Islander/Native Hawaiian	605	6.0
Multiple	619	6.2
Missing	4,613	46.1
**Living situation**		
Stable housing	8,585	88.2
Foster care	591	6.1
Juvenile justice facility	357	3.7
Unstable housing	201	2.1
**Ever had vaginal and/or anal sex**		
No	7,039	71.2
Yes	2,851	28.8

*Note*: Except for race, percentages are based on those with non-missing data

### Perceived barriers

Table 3 shows perceptions of each barrier and burden of five or more barriers in the sample as a whole, and differences in perceptions by participant characteristics. About half of the youth reported concerns about test results (52.7%), cost (52.0%), and confidentiality (49.8%). Two other common barriers were related to interacting with providers, including discomfort talking with staff about sexual health (36.7%) and worrying about being judged by staff (36.4%). Youth were less likely to report lack of knowledge about the visit (29.1%) and lack of access (26.3%) as barriers to SRH services. Almost one fifth of youth (17.8%) reported five or more barriers.

**Table 3:** Percent of participants who perceive given barriers to accessing sexual and reproductive health services

	***Group*** ***n***	Test results	Cost of service	Confidentiality of service	Discomfort talking with staff	Judgment by staff	Lack of knowledge about visit	Lack of access	5 or more Barriers
**All participants**	10,015	52.7	52.0	49.8	36.7	36.4	29.1	26.3	17.8
**Gender**									
Male (reference)	5,166	51.1	49.4	43.9	35.1	30.1	27.1	22.5	13.3
Female	4,664	54.5**	54.4***	56.1***	37.9*	42.8***	31.1***	29.8***	22.3***
Transgender/non-binary/multiple genders	129	53.9	68.8***	59.2**	49.2**	53.5***	34.1	42.5***	32.6***
**Age**									
10-14 years (reference)	4,579	56.3	54.9	53.8	43.8	40.2	33.9	30.8	21.8
15-19 years	5,369	49.6***	49.4***	46.4***	30.6***	33.2***	24.9***	22.4***	14.5***
**Sexual orientation**									
Straight/heterosexual (reference)	8,140	51.9	50.7	48.7	36.3	34.8	28.5	25.3	16.6
LGBQ+	1,503	57.0***	59.2***	56.7***	37.5	44.7***	31.1*	31.0***	25.1***
**Ethnicity/race**									
Hispanic (reference)	7,546	53.0	52.6	50.6	35.9	36.7	27.9	25.8	17.6
Non-Hispanic White	968	46.8**	51.3	46.5	41.2*	36.0	34.4**	29.7	19.1
Non-Hispanic Black	479	47.8	39.5***	40.9**	30.8	28.9*	23.5	18.3**	11.1**
Non-Hispanic Asian or Pacific Islander/Native Hawaiian	407	65.8***	62.4**	54.9	44.0*	44.4*	38.4***	32.3	29.7***
Non-Hispanic American Indian/ Alaska Native	133	58.6	44.6	42.9	42.3	40.5	36.4	32.8	17.3
Non-Hispanic Multiple	331	52.6	45.8	48.1	33.5	29.5	29.4	27.2	13.9
**Living situation**									
Stable housing (reference)	8,585	52.6	53.0	51.3	37.0	36.9	29.5	26.8	18.5
Foster care	591	57.5	50.9	46.7	34.8	37.5	24.6	25.3	15.9
Juvenile justice facility	357	43.2**	31.0***	22.5***	22.8***	19.9***	22.1*	11.0***	5.6***
Unstable housing	224	53.4	55.9	47.5	42.0	45.9	31.4	31.7	17.4
**Ever had vaginal and/or anal sex**									
No (reference)	7,039	54.8	55.0	53.1	40.5	38.7	31.8	28.7	20.3
Yes	2,851	47.9***	44.7***	41.9***	26.5***	30.7***	22.5***	20.0***	11.8***

*Note*: Cell entries are the percent who agree. Asterisks indicate statistically significant difference compared to reference group (* *p* < 0.05, ** *p* < 0.01, *** *p* < 0.001)

**Fig. 1:**
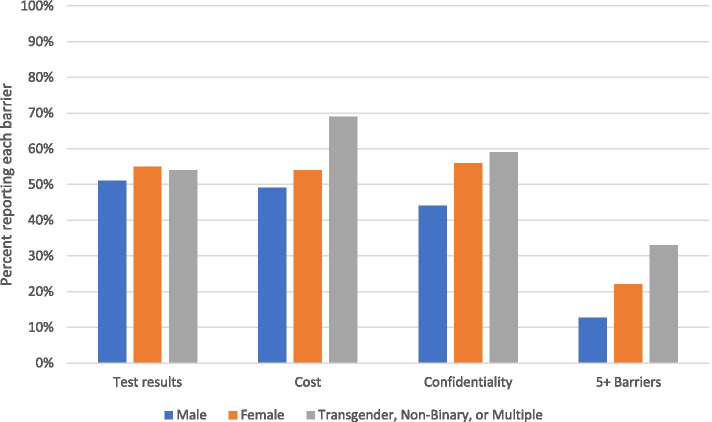
Perceived barriers to SRH services, by gender

**Fig. 2:**
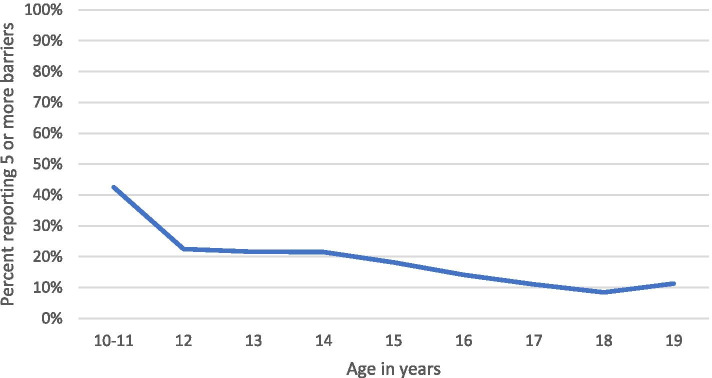
Burden of five or more barriers, by age

Youth of different gender identities, sexual orientation, and age groups consistently had differences in perceptions of barriers. Compared to male youth, female youth were significantly more likely to perceive every barrier and five or more barriers (Fig. 1). Similarly, youth identifying as transgender, non-binary, or with multiple genders were significantly more likely than male youth to report five or more barriers and every barrier except worry about test results and not knowing what to expect during a clinic visit. Compared to heterosexual youth, LGBQ+ youth were more likely to perceive every barrier except discomfort talking with staff. Younger youth were significantly more likely than older youth to perceive every barrier and five or more barriers. Figure 2 illustrates the decline in burden of five or more barriers by age.

Reported barriers were more mixed across categories of ethnicity/race. Because the majority of our sample identified as Hispanic or Latino, perceptions of barriers for this group closely mirror the percentages already reported for the sample as a whole. That is, over half of Hispanic youth reported concern about test results, cost of service, and confidentiality, and almost one-fifth reported five or more barriers. Hispanic youth were more likely to report cost of service and confidentiality as barriers, and less likely to report discomfort talking with staff and not knowing what to expect in a clinic visit compared to Non-Hispanic youth (data not shown). Asian or Pacific Islander/Native Hawaiian youth were the only group to have consistently higher percentages reporting barriers than the Hispanic reference group. Black youth were the only group to have consistently lower percentages reporting barriers than the Hispanic reference group. Youth living in juvenile justice facilities were also less likely to report all barriers, including five or more barriers, compared to youth living in stable housing. This pattern was also similar for those who reported having vaginal and/or anal sex, as they were less likely to report all barriers as well as for five or more barriers compared to youth who did not have vaginal and/or anal sex (see Table 3).

### Participant characteristics and perceived barriers

Results for logistic regressions of all the participant characteristics on the three most common barriers and for five or more barriers are shown in Table 4. When controlling for all other participant characteristics, females and youth identifying as transgender/non-binary/multiple genders had higher odds of perceiving cost of service and confidentiality of service as barriers as well as five or more barriers, with 1.10-1.68 times the odds for females and 1.51-2.38 times the odds for youth identifying as transgender/non-binary/multiple genders compared to males.

**Table 4:** Predictors of perceiving barriers to sexual and reproductive health services

	Test results (*N* = 9,008)		Cost of service (*N* = 9,036)		Confidentiality of service (*N* = 9,013)		5 or more barriers (*N* = 9,167)	
	OR (95% CI)	*p*	OR (95% CI)	*p*	OR (95% CI)	*p*	OR (95% CI)	*p*
**Gender**								
Male	Reference	—	Reference	—	Reference	—	Reference	—
Female	1.05 (0.96, 1.15)	0.266	1.10 (1.00, 1.20)	0.045	1.48 (1.35, 1.61)	< 0.001	1.68 (1.49, 1.88)	< 0.001
Transgender/non-binary/multiple genders	0.82 (0.56, 1.21)	0.316	1.89 (1.24, 2.88)	0.003	1.51 (1.01, 2.24)	0.044	2.38 (1.55, 3.63)	< 0.001
**Age** (continuous)	0.90 (0.87, 0.93)	< 0.001	0.96 (0.93, 0.99)	0.016	0.90 (0.87, 0.93)	< 0.001	0.84 (0.81, 0.88)	< 0.001
**Sexual orientation**								
Straight/heterosexual	Reference	—	Reference	—	Reference	—	Reference	—
LGBQ+	1.21 (1.07, 1.37)	0.002	1.36 (1.20, 1.54)	< 0.001	1.24 (1.10, 1.41)	0.001	1.42 (1.22, 1.64)	< 0.001
**Ethnicity/race**								
Hispanic	Reference	—	Reference	—	Reference	—	Reference	—
Non-Hispanic	—	—	—	—	—	—	—	—
White	0.76 (0.65, 0.88)	< 0.001	0.91 (0.78, 1.06)	0.238	0.82 (0.70, 0.96)	0.012	1.01 (0.83, 1.23)	0.899
Black	0.88 (0.72, 1.08)	0.224	0.65 (0.53, 0.81)	< 0.001	0.77 (0.62, 0.95)	0.015	0.66 (0.48, 0.90)	0.009
Asian/Pacific Islander/ Native Hawaiian	1.68 (1.34, 2.11)	< 0.001	1.37 (1.09, 1.72)	0.006	1.14 (0.91, 1.42)	0.262	1.82 (1.42, 2.33)	< 0.001
American Indian/Alaska Native	1.28 (0.87, 1.89)	0.213	0.76 (0.52, 1.12)	0.167	0.73 (0.50, 1.08)	0.119	1.02 (0.62, 1.68)	0.930
Multiple	1.01 (0.79, 1.28)	0.954	0.75 (0.59, 0.95)	0.018	0.93 (0.73, 1.18)	0.547	0.69 (0.49, 0.98)	0.036
**Living situation**								
Stable housing	Reference	—	Reference	—	Reference	—	Reference	—
Foster care	1.27 (1.05, 1.53)	0.013	1.02 (0.84, 1.23)	0.854	0.92 (0.76, 1.11)	0.378	0.85 (0.66, 1.10)	0.210
Juvenile justice facility	0.87 (0.68, 1.11)	0.260	0.58 (0.45, 0.76)	< 0.001	0.42 (0.32, 0.55)	< 0.001	0.47 (0.29, 0.76)	0.002
Unstable housing	1.13 (0.83, 1.53)	0.430	1.41 (1.04, 1.91)	0.027	1.12 (0.83, 1.52)	0.450	1.06 (0.71, 1.58)	0.768
**Ever had vaginal and/or anal sex**								
No	Reference	—	Reference	—	Reference	—	Reference	—
Yes	0.93 (0.84, 1.04)	0.216	0.80 (0.71, 0.89)	< 0.001	0.91 (0.82, 1.02)	0.101	0.78 (0.67, 0.91)	0.001

*Note*: Odds ratios are based on mixed-effects logit models, accounting for the nested structure of the data (participants within cohorts and cohorts within agencies)

Older youth had lower odds of perceiving any of the barriers, including five or more barriers, compared to younger participants. For every year of increase in age, there was a 0.04-0.16 decrease in odds of reporting test results, cost, and confidentiality as barriers and five or more barriers. Youth identifying as LGBQ+ had 1.21-1.42 times the odds of reporting test results, cost, and confidentiality as barriers as well as five or more barriers, relative to heterosexual youth.

Compared to Hispanic youth, Asian or Pacific Islander/Native Hawaiian youth had 1.37-1.80 times the odds of perceiving test results and cost as barriers as well as perceiving five or more barriers. In contrast, Black youth had 0.65-0.77 times the odds of perceiving cost and confidentiality as barriers as well as reporting five or more barriers. The vast majority (88.3%) of youth who identified as American Indian/Alaska Native also identified as Hispanic and/or multiple races. For this reason, we conducted an additional analysis in which any youth who identified as American Indian/Alaska Native (*n* = 1,135) was coded as such, regardless of any other identification. Most results for the subsequent logistic regression were unaffected, except that this more inclusively-defined group of American Indian/Alaska Native youth had 0.82-0.83 times the odds of perceiving confidentiality and discomfort talking with staff as barriers than Hispanic youth.

Compared to the reference group of youth living in stable housing, youth living in juvenile justice facilities had 0.42-0.58 times the odds of perceiving cost and confidentiality as barriers, as well as reporting five or more barriers. The only statistically significant difference for youth in other living situations was youth in foster care had 1.27 times the odds of perceiving test results as a barrier and unstably housed youth had 1.41 times the odds of perceiving cost as a barrier. Youth with sexual experience had 0.78-0.80 times the odds of perceiving cost as a barrier, as well as reporting five or more barriers.

The pattern of results was similar for the four barriers reported by fewer than half of adolescents (see Supplemental Table 1). The odds of reporting concerns about judgment by staff and lack of access were significantly higher for females and for youth identifying as transgender/non-binary/multiple genders than for males. When controlling for all other participant characteristics, increasing age was associated with decreased odds of perceiving each of these four barriers. The odds of reporting concerns about judgment by staff and lack of access were higher for LGBQ+ youth than for heterosexual youth. Compared to Hispanic youth, Asian or Pacific Islander/Native Hawaiian youth had the highest odds of reporting discomfort talking with staff, judgment by staff, and lack of knowledge about the visit as barriers. In contrast, Black youth had lower odds of reporting judgment by staff and lack of access as barriers. Living in a juvenile justice setting was associated with lower odds of reporting judgment by staff and lack of access as barriers. Sexual experience was associated with lower odds of reporting discomfort talking with staff and lack of knowledge about the visit as barriers.

## Discussion

This study found that concerns about test results, cost of services, and confidentiality were the most common barriers to SRH services perceived by adolescents prior to participating in a sexual health education program in California. Across these barriers, younger adolescents consistently reported greater perceived barriers, even after controlling for sexual experience and other variables. Early adolescence represents a critical developmental stage that requires support in cultivating autonomy and decision-making skills, particularly around SRH issues [35, 36]. Similarly, when assessing the burden of five or more barriers, females, younger adolescents, LGBTQ+ youth, and Asian or Pacific Islander/Native Hawaiian youth all had greater odds of reporting five or more barriers, indicating that some groups may face a higher overall burden to access. Differing from previous research, after controlling for other characteristics, Black youth had lower odds of reporting many of the barriers, as well as of reporting five or more barriers [12, 13, 33].

Consistent with Penchansky and Thomas’s dimensions of access to health care, about half of the respondents were concerned about cost, test results, and confidentiality, which align with the affordability and accommodation dimensions in the original theory, while accessibility was a concern to about a quarter of the respondents. Our results show that lack of knowledge of what to expect in a clinic visit was a barrier to over a quarter of youth (29.1%). Although the original theory did not mention awareness as a dimension, subsequent research has recommended this addition [32], which is supported by our results.

These results highlight the need to address disparities among certain groups of adolescents. For example, youth in foster care, who are much more likely to experience pregnancy in adolescence, may face the additional barrier of judgmental guardians and require policies and trauma-informed programs tailored to their needs [37]. In contrast, youth in juvenile justice facilities generally reported lower barriers to services, which may reflect on-site access to SRH care [33]. In addition, sexual health education and clinical services need to be responsive to and inclusive of diverse genders and sexual orientations. Differences by race and ethnicity also show the need for programs and services that are culturally appropriate. The higher perceived barriers among youth identifying as Asian or Pacific Islander/Native Hawaiian require efforts to address stigma and confidentiality concerns in these communities [16].

These results also highlight quantitatively the burden of multiple barriers to SRH care for adolescents. Previous qualitative research with adult women seeking abortions suggests that the effects of barriers are cumulative, with women who simultaneously face multiple barriers experiencing worse outcomes [21]. Other qualitative research suggests that the problem of multiple barriers may be compounded for youth from marginalized groups, who are more likely to forgo care when they face discriminatory treatment in the healthcare system on top of barriers unrelated to discrimination [38]. Thus, to reduce disparities in health outcomes, it is vital to improve access for vulnerable groups that may face a greater burden of barriers [22].

While this study focused on barriers to access, other research has identified enablers or facilitators that increase the likelihood that adolescents will seek services. Parental communication about SRH, social norms, and individual motivation are all potential facilitators [39, 40]. Providing more information to adolescents about available services can increase awareness and may alleviate concerns about certain barriers. One potential avenue for this information is through sexual health education in schools and other settings. CA PREP requires all agencies implementing sexual health education to provide information about local SRH services [41]. Sexual health education programs can directly address adolescents’ questions and concerns, such as discussing practices to ensure privacy and confidentiality in clinics, as well as providing local clinic information including available services, hours, location, and cost [42]. Schools may also consider developing formal referral systems to SRH services or providing contraception at school-based health centers [43, 44].

Clinics also must better address youth concerns. Previous studies of youth-friendly services have identified the importance of ensuring confidentiality, respectful interactions with providers and other staff, specialized provider training, and addressing other logistical barriers such as insurance and cost [45, 46]. Programs and policies should also consider broader structural and social determinants of health, such as educational and employment opportunities, which may affect adolescents’ need for and access to services.

### Limitations

Although CA PREP prioritizes services in low-income communities, this study did not assess other social determinants of health, including household socioeconomic status, insurance status, and geographic location, which other studies have shown to be associated with access to care as well as other adolescent health outcomes [17, 47]. Because the study was based in California, which mandates sexual health education and confidential access to care for minors, these results may underestimate the access barriers that youth face in other states. Some barriers may have been interpreted differently by respondents. For example, the question on confidentiality did not distinguish between privacy concerns with peers and confidentiality concerns with parents. Similarly, worry about test results may be interpreted as worry about what would happen if a test is positive or how this information is communicated.

Traditional measures of race and ethnicity do not align with many respondents’ self-identification, particularly those who identify as Hispanic or Latino. To avoid losing over 4,500 Hispanic youth in multivariate analyses due to missing race, we aggregated race and ethnicity. However, this meant that the 40.9% of youth who *did* report a race in addition to their Hispanic ethnicity were not represented in the racial categories. Follow-up analyses indicated that this did not seriously affect results for American Indian/Alaska Native youth, the group most affected by this aggregation.

## Conclusion

The majority of adolescents participating in sexual and reproductive health education programs face barriers to accessing appropriate SRH services, with youth in certain demographic groups including younger adolescents, youth identifying as transgender/non-binary/multiple genders, LGBQ+ youth, and Asian or Pacific Islander/Native Hawaiian youth more likely than other youth to report barriers. Access can be improved through strengthening linkages between clinics and sexual health education programs, providing youth-friendly clinical services, and ensuring youth have sufficient information, skills and support to access care.

## Supplementary Information

**Additional file 1: Supplemental Table 1.** Predictors of perceiving other barriers to SRH services

## Data Availability

The dataset analyzed for the current study is not publicly available due to California state data regulations and concerns about confidentiality for study participants. However, the dataset is available from the authors upon reasonable request and subject to the approval of the California Department of Public Health - Maternal, Child and Adolescent Division.

## Abbreviations

CA: California
CDPH: California Department of Public Health
HIV: Human immunodeficiency virus
MCAH: Maternal, Child and Adolescent Health
LGBQ+: Lesbian, gay, bisexual, queer, and other sexual orientations
LGBTQ: Lesbian, gay, bisexual, transgender, and queer
OR: Odds ratio
PREP: Personal Responsibility Education Program
SRH: Sexual and reproductive health
STI: Sexually transmitted infection
