# Supplementary material for: Adolescents’ perceived barriers to accessing sexual and reproductive health services in California: a cross-sectional survey
Source: BMC Health Serv Res. 2021 Nov 22;21:1263. doi: 10.1186/s12913-021-07278-3 (PMC8609799; doi:10.1186/s12913-021-07278-3)
Supplement: Supplementary file 1 — Additional file 1: Supplemental Table 1. Predictors of perceiving other barriers to SRH services [file 12913_2021_7278_MOESM1_ESM.docx]

**Supplemental Table. Predictors of perceiving other barriers to SRH services**

|  | **Discomfort talking with staff**  (*N* = 9,033) | | **Judgment by staff**  (*N* = 9,009) | | **Lack of knowledge**  **about visit**  (*N* = 9,028) | | **Lack of access**  (*N* = 9,034) | |
| --- | --- | --- | --- | --- | --- | --- | --- | --- |
|  | OR (95% CI) | *p* | OR (95% CI) | *p* | OR (95% CI) | *p* | OR (95% CI) | *p* |
| **Gender** |  |  |  |  |  |  |  |  |
| Male | Reference | — | Reference | — | Reference | — | Reference | — |
| Female | 1.09 (1.00, 1.20) | 0.062 | 1.62 (1.48, 1.78) | < 0.001 | 1.17 (1.06, 1.29) | 0.002 | 1.35 (1.22, 1.50) | < 0.001 |
| Trans/non-binary/multiple genders | 1.79 (1.21, 2.65) | 0.004 | 1.95 (1.32, 2.88) | 0.001 | 1.29 (0.86, 1.95) | 0.221 | 2.04 (1.36, 3.06) | 0.001 |
| **Age** (continuous) | 0.81 (0.79, 0.84) | < 0.001 | 0.89 (0.86, 0.92) | < 0.001 | 0.84 (0.81, 0.87) | < 0.001 | 0.84 (0.81, 0.87) | < 0.001 |
| **Sexual orientation** |  |  |  |  |  |  |  |  |
| Straight/heterosexual | Reference | — | Reference | — | Reference | — | Reference | — |
| LGBQ+ | 0.95 (0.84, 1.08) | 0.438 | 1.30 (1.15, 1.47) | < 0.001 | 1.05 (0.91, 1.19) | 0.517 | 1.17 (1.02, 1.34) | 0.025 |
| **Ethnicity/race** |  |  |  |  |  |  |  |  |
| Hispanic | Reference | — | Reference | — | Reference | — | Reference | — |
| Non-Hispanic | — | — | — | — | — | — | — | — |
| White | 1.12 (0.96, 1.31) | 0.144 | 0.89 (0.76, 1.04) | 0.135 | 1.25 (0.06, 1.47) | 0.008 | 1.17 (0.98, 1.39) | 0.075 |
| Black | 0.90 (0.72, 1.12) | 0.337 | 0.73 (0.58, 0.91) | 0.006 | 0.82 (0.64, 1.04) | 0.099 | 0.64 (0.49, 0.84) | 0.001 |
| Asian or Pacific Islander/Native Hawaiian | 1.28 (1.03, 1.61) | 0.029 | 1.36 (1.09, 1.70) | 0.007 | 1.49 (1.18, 1.87) | 0.001 | 1.22 (0.96, 1.55) | 0.109 |
| American Indian/Alaska Native | 1.30 (0.88, 1.91) | 0.190 | 1.04 (0.70, 1.54) | 0.857 | 1.39 (0.93, 2.09) | 0.109 | 1.32 (0.87, 2.00) | 0.189 |
| Multiple | 0.84 (0.65, 1.08) | 0.177 | 0.64 (0.49, 0.83) | 0.001 | 0.98 (0.76, 1.28) | 0.885 | 0.99 (0.75, 1.30) | 0.917 |
| **Living situation** |  |  |  |  |  |  |  |  |
| Stable housing | Reference | — | Reference | — | Reference | — | Reference | — |
| Foster care | 0.94 (0.77, 1.15) | 0.563 | 1.09 (0.90, 1.32) | 0.393 | 0.73 (0.58, 0.90) | 0.004 | 0.87 (0.70, 1.09) | 0.220 |
| Juvenile justice facility | 0.81 (0.60, 1.08) | 0.143 | 0.63 (0.47, 0.85) | 0.002 | 0.99 (0.74, 1.32) | 0.930 | 0.42 (0.29, 0.62) | < 0.001 |
| Unstable housing | 1.53 (1.12, 2.09) | 0.007 | 1.68 (1.24, 2.28) | 0.001 | 1.10 (0.79, 1.54) | 0.558 | 1.31 (0.94, 1.83) | 0.108 |
| **Ever had vaginal and/or anal sex** |  |  |  |  |  |  |  |  |
| No | Reference | — | Reference | — | Reference | — | Reference | — |
| Yes | 0.72 (0.64, 0.81) | < 0.001 | 0.90 (0.80, 1.01) | 0.077 | 0.80 (0.71, 0.91) | 0.001 | 0.90 (0.79, 1.02) | 0.098 |

Note: Odds ratios are based on mixed-effects logit models, accounting for the nested structure of the data (participants within cohorts and cohorts within agencies).
